# Notes and Comments

**Published:** 1884-03

**Authors:** 


SOMETHING NEW IN BLOOD-LETTING.—A writer in the *British Medical Journal* had a patient over 50 years of age, who fell into a stupor, with high arterial action and distended venous blood vessels. He proposed bleeding, but the attendants were horrified. He then took an aspirator and quietly introduced it into the left jugular vein, which was much distended, and four ounces of blood were taken. In half an hour the result was so satisfactory that the operation was repeated and six ounces more taken. She soon recovered, but neither she nor her nervous friends had any idea that she had been bled until the process was subsequently explained to them.—*Pacific Medical and Surgical Journal.*

REMARKABLE FECUNDITY.—H Algernon Hodson, of Brighton, writes to the *Lancet*, December 15th: "You may think the following case worth publishing, as one can but hope it to be rather an unusual one. A patient of mine gives me the following history: She was married when twenty-six years of age, and ceased menstruating when thirty-nine, after the birth of her last child, having had sixteen children, and one miscarriage in fourteen years. She nursed each one of her children, but found the nursing did not in the least prevent her becoming pregnant. The dates of birth were July, 1845, a son; November, 1846, a son; February, 1848, a son; November, 1849, twin daughters; September, 1851, twins (son and daughter); November, 1852, daughter; November, 1853, twins; July, 1854, twins (son and daughter); July, 1855, twins (sons); 1856, miscarriage; May, 1857, daughter; June, 1858, son. She is a tall, spare woman, and at the present time is in very fair health. She is sixty-three years of age. Out of all her children only six are now living.—*Maryland Medical Journal.*

A death from "soothing syrup" having occurred in Brooklyn, the coroner's jury have very sensibly recommended the passage of a law by the Legislature, forbidding the sale of such preparations, except on physicians' prescriptions.—*Gaillard's Journal.*

BARTHOLDI'S GREAT STATUE.—A very large and beautifully executed picture of Bartholdi's great statue of "Liberty Enlightening the World" has been presented to us by the Travelers' Insurance Company, of Hartford, Conn., who have been among the most liberal contributors to the fund. The picture, which is 26x36 inches in size, gives an excellent idea of the superb work of art which is to adorn the harbor of New York.

### Notes and Comments.

---

Nitro-Glycerin and the Chloride of Gold and Sodium in the Treatment of Albuminuria.—The following is from a paper read by Dr. Bartholow before the Philadelphia County Medical Society:

Hitherto the therapeutics of renal diseases have not advanced in the same ratio as our knowledge of their pathology. It cannot be said now that a cure has been found, but that two remedies of real value are available. My contribution to this symposium on albuminuria consists in an attempt to define the place which these remedies should occupy in a curative scheme. To do this, in even the briefest way, I must clear the ground with a preliminary statement.

I start with the proposition that those renal lesions united by the common symptom—albuminuria—are of neural origin. There is a kinship between diabetes and Bright's disease. One of these is sometimes substituted for the other; and during the course of some rare cases of exophthalmic goitre this substitution occurs. Irritation of a certain part of the floor of the fourth ventricle is followed by glycosuria; of another part by albuminuria. The recent observations of Da Costa and Longstreth prove that a relation exists, whether casual or sequential, between certain renal lesions and degenerative changes in some ganglia of the abdominal sympathetic. The hypertrophy of the muscular coat of the arterioles, discovered by Dr. George Johnson, and the increased tension of the vascular system due to an irritation of the vaso-motor center in the medulla, both present in the chronic forms of albuminuria, are further evidences of the agency of the nervous system. It was, more especially, the condition of elevated tension of the vessels which led to the use of nitro-glycerin. This remedy before all else reduces the vascular tension. It also lessens the work of the heart by removing the inhibition exercised by the pneumo-gastric nerve.

This remedy appears to have been first used by Mr. Robson, an English surgeon, in cases of albuminuria, and by him employed, because the high tension of the vascular system has proved to be so pronounced an element in the more chronic cases. I have myself seen some remarkable instances of relief—indeed of cure—effected by it. If time were now available, I could give some striking examples. In cases of mitral disease, accompanied by albuminuria, it also renders the highest service, for the diminished peripheral tension lessens the work to be done by the heart, and assists in the more equal distribution of the blood. The effect of this in relieving the renal congestion is obvious.

Chloride of gold and sodium has quite another function. It has long been known that this remedy has a special direction to the genito-urinary apparatus. The ovarian and uterine organs in the female, the testes and vesiculæ seminales in the male, are stimulated by it, and the kidneys, by means of which it is eliminated, and in which it tends to accumulate, are decidedly affected by it in function and structure. In common with some other agents of the class to which gold belongs—for example, corrosive sublimate—the chloride acts on connective tissue and checks its over-production, or its hyperplasia. It would be quite impossible in this note to go over the evidence on these points, and hence I must ask your assent to these statements. They have been accepted as true of gold, from the days of the alchemists and iatro-chemists, as any one may ascertain from that curious collection of mediæval medical learning—the "Anatomy of Melancholy." It has happened, strangely enough, that Hahnemann and his followers have profitted by this knowledge, and have used gold preparations—especially *aurum potabile*—in the treatment of renal diseases with success.

How and when are these remedies to be used?

Nitro-glycerin is now administered, as all present know, in the form of the centesimal solution—1 minim of the pure drug to 100 minims of alcohol. The initial dose of this one per cent. solution is one minim, which should be increased until the very characteristic physiological effects are produced. The susceptibility to the action of nitro-glycerin varies greatly, and hence the dose cannot be stated in advance. It is necessary to produce some obvious effect. To maintain the same level of action, a slight increase in the dose may be required from time to time. As the effect is not lasting, the interval between the doses should not exceed three or four hours.

The administration of nitro-glycerin should begin in acute cases immediately after the subsidence of the acute symptoms. It is indicated in chronic cases at all periods, but is more especially useful if given before hypertrophy of the muscular layer of the arterioles has taken place. When it acts favorably, the amount of albumin in the urine steadily diminishes. The mechanism of its action consists in the lowering of the pressure in the renal vessels. How far any curative effect proceeds from action of this remedy on the sympathetic system remains to be determined.

Chloride of gold and sodium is indicated in the subacute and chronic cases, especially the latter. The earlier it is given the better, if structural changes are to be prevented or arrested. The good effects to be expected from it will depend necessarily on the extent of the damage already inflicted on the kidneys.

The usual dose is $\frac{1}{26}$ grain twice a day, but this may be much increased if necessary. At the outset, $\frac{1}{10}$ grain may be given; in a week the dose should be lowered to $\frac{1}{15}$ grain, and after a month the regular dose of $\frac{1}{20}$ grain should be steadily pursued, with occasional intermissions. Indigestion, gastralgia and colic pains, nausea or diarrhœa, are occasionally caused by it; and, if so, the quantity administered must be reduced. It is usually borne without any discomfort, but, after prolonged administration, salivation, weakness, emaciation, trembling, and other nervous phenomena, may occur possibly. Such effects, however, are wanting in my experience.

The treatment of albuminuria by nitro-glycerin and the chloride of gold and sodium does not necessitate the exclusion of other means—hygienic, climatic or dietetic. These remedies should, however, be given uncombined at different hours, and their actions should not be hindered or obscured by the effects of other agents given with like purpose. To this general statement there may be two exceptions: with nitro-glycerin, amyl nitrite or sodium nitrite may be given; with the gold-and-sodium chloride, corrosive sublimate may be combined. If doubts may be felt in regard to the propriety of depending on the utility of these remedies, they need not be long experienced, for, if no good effects are observed in two weeks, they may then be discontinued.—*New York Medical Journal, February 2d.*

DR. HERBERT JUDD, Galesburg, Ill., reports the following case in the *Journal* of the American Medical Association: February 5, 1883, at 8 P. M., I was called to see C. H., at the Union Hotel, who, while descending the stairs of the hotel one hour before, had slipped and fallen upon his left side and back. His general condition was good, his habits temperate. The only treatment I advised was to apply over the seat of pain a belladonna plaster. The plaster was to be cut from a pattern I made of paper, triangular in shape, and has since been found to aggregate about twenty-six square inches in surface; the plaster was of the ordinary roll plaster, prepared by Seabury & Johnson, not porous. There had been no liniment nor any irritating or soothing lotion of any kind used. There was no abrasion of the skin. At 11 o'clock, three hours later, I was telephoned in great alarm and distress by the hotel people to hurry to my patient. Their replies to my questions concerning his condition were such that I queried with Dr. Ira Wilcox, of New York city (then a guest at my house), and Drs. Hopper and Edgerton, who were also present, if it were possible the patient was affected by the plaster; the plaster had been cut and brought to me for inspection before it was applied, as it had at first been cut wrong and would not fit the body as I had directed; in this manner the size of the plaster and the case had been brought to the notice of the above named physicians. We all thought it could not be the plaster.

I found the patient suffering in an extreme degree from the poisonous effect of belladonna; at least, such was my opinion and explanation to the terrified attendants. The patient could not see distinctly; the face and neck were flushed to a deep scarlet; he could not swallow and was in spasm. I removed the plaster and gave a hypodermic of morphine; he immediately became quiet, and at the end of one hour was in a nearly natural sleep. The disturbance of vision lasted three days. My patient was a member of the traveling public and a favorite among them, also an old resident of this city, so that I became *famous* for not only giving *strong medicine*, but for using *strong* belladonna plaster.

---

*Drs. Taliaferro & Noble.*—We desire to call attention to the advertisement of this firm, to be found on our first advertising page. The reputation of these gentlemen in their specialty is too well known to need any words of commendation at our hands.

A BAD PRECEDENT.—A bad precedent has been established by the residents of a small town near Denver, Col. It seems that a man by the name of Eli Madlong, pretending to be a physician, though he had never received a medical education, prescribed some medicine for a patient who died, presumably from the effects of the prescription. Whereupon the friends of the deceased, being indignant, hanged the venturesome practitioner by the neck until he was as dead as his unfortunate patient. It is not stated how the mob knew that death was the result of the medicine; it is quite evident that experts were not called in to testify; if there had been they would have shown that the medicine, instead of doing harm, actually prolonged the patient's life. If every doctor whose patient dies shortly after taking medicine is to be hanged, who of us is safe? Perhaps, after all, this is the true solution of the way to elevate the standard of medical education; if a practitioner knows that he is in danger of being hung unless he can prove his ability as a physician, it is probable he will take care to qualify himself before he begins the practice of medicine. In the meanwhile, if every unqualified doctor is to be hung, there is danger of a bull movement in hemp.—*Medical Review.*

---

ANTISEPTIC SURGERY.—Says the *Medical News* (Dec. 8th): If additional proof of the value of antiseptic over old methods of managing wounds were needed, it would be found in the results obtained by Professor Bruns, of Tubingen, and published in a recent brochure. Of one hundred and forty-nine amputations performed between 1877 and 1882, only 5.5 per cent. proved fatal. During that period, amputation of the leg was attended with a mortality of 3.2 per cent., and of the thigh of 2.5 per cent. From 1843 to 1863, or in preantiseptic days, the same operations showed, respectively, a mortality of 29.7 and 43.5 per cent.

Equally striking results were obtained by Professor Esmarch, of Kiel, in 92 resections of the knee done between 1857 and 1882, of which a full account may be found in an inaugural dissertation by Dr. Mensing. Of 21 cases treated in accordance with old methods, 14 recovered, and 7, or 33.3 per cent., perished. Of 23 cases managed with Lister's dressing, 21 recovered, and 2, or 8.7 per cent., died. Of 48 cases treated with the antiseptic dressing of Neuber, 47 recovered, and 1, or 2 per cent., perished. In other words, the mortality of antiseptic resections was 4.2 per cent. as against 33.3 per cent. or non-antiseptic operations.

DR. CALEB WINSLOW (*Maryland Medical Journal*) gives a summary of ninety-nine cases of lithotomy with only one death. Most of the patients operated upon were natives of eastern North Carolina, living in that portion of territory north of the Albemarle Sound and east of the Chowan river, a tract about sixty miles long and thirty broad. This country is flat and swampy, and would seem to be free from any geological conditions which would be likely to favor the production of stone. The habits of the people present no especial peculiarity in regard to food or mode of life, and the affection occurs with equal frequency amongst the rich and the poor.

Dr. Agnew states specifically that stone in the bladder is not common in North Carolina, while Dr. Gross leaves it out of his list of States in which it is of frequent occurrence. So many cases coming under the notice of one practitioner living in a sparsely settled country, in a period of eighteen years, is rather remarkable, and would indicate a marked prevalence of the affection in that section of the State.

---

IMMEDIATE TREATMENT OF FRACTURES BY PLASTER OF PARIS SPLINTS.—Mr. John Croft, in the *British Medical Journal*, has this to say in regard to the immediate treatment of fractures by plaster of Paris splints: Mr. Battle, the Registrar of St. Thomas' Hospital, has kindly ascertained for me that, during 1881 and 1882, 403 fractures were treated in this way, and none but good results ensued. No instances of gangrene, or bad or delayed union, or splint sores, occurred. This number, with that already reported, viz., 498, makes up a total of 901 cases treated with the best results.—*Braithwaite's Retrospect.*

---

THE AMERICAN MEDICAL ASSOCIATION.—The thirty-fifth annual session will be held in Washington, D. C., on Tuesday, Wednesday, Thursday and Friday, May 6th, 7th, 8th and 9th, 1884, commencing on Tuesday at 11. a. m.

### DANGER FROM QUININE.

Our exchanges have considerable to say just at present about the dangers that may exist in the administration of quinine, and the injurious results that sometimes follow its use.

While it is well to utter words of caution, yet it seems almost like a waste of time to caution physicians that quinine may be, in certain cases and under certain circumstances, a dangerous article; for we take it that every ordinarily well-informed medical man is thoroughly aware of this fact.

We know that quinine is one of the most valuable drugs in our possession when properly and intelligently used; and we also know that it is no exception to the rule, which holds good with all drugs, that if used when not indicated, or in a wrong way, it is capable of working harm.

If, for instance, we desire to give quinine for its tonic effects only, and we order five or ten grains thrice daily, we will do more harm than good; while, on the other hand, if we endeavor to control a well-marked intermittent fever with one or two grains, our experience will be similar to that.—*Medical and Surgical Reporter.*

---

ANTISEPSIS IN OVARIOTOMY AND BATTEY'S OPERATION.—Dr. Robert Battey, of Rome, Ga., in an article on this subject to the *Virginia Medical Monthly*, has this to say:

Of the spray and the use of carbolic acid in general, whilst I think it has been pretty clearly shown by Keith, Bantock, and Tait, that neither is essential to the highest success, and when strong, may even prove poisonous to patient and surgeon, I feel assured that weaker solutions do no harm, and think they may serve to guard the patient against any slight imperfections in the details of cleansing. Quite sure am I that my own results, with the acid and the spray, are now as good as I could desire. Let those who can get the same results without these aids do so; for myself, I am content to hold them as valuable assistants until their utter uselessness has been more conclusively shown.

---

THIS number of THE JOURNAL does not make its appearance as was promised in our prospectus. This delay has been unavoidable. In future we hope to be out on time.

PATRICK B. TULLY, a new Representative in Congress from California, has introduced in the House a bill refusing the privileges of the mails to the advertisements "of any medical preparation, compound, or prescription, or any punch, bitters, cordial, or similar compound or preparation to be used as medicine or mixed with food, liquor, wine, or other substance used as a beverage or as food or medicine," unless the manufacturers thereof can procure from the Federal Patent office a certificate that it is not noxious or dangerous to health. The progress of this bill will be watched with interest, but it will doubtless be beset with many difficulties, so that prognosis for its survival should be very guarded.—*Boston Medical and Surgical Journal.*

---

VICARIOUS MENSTRUATION.—A case is related (*Progres Medical*) of a girl who received a blow behind the right ear when six years old, which was followed by a yellowish discharge which has since continued. At the age of fourteen she was awakened one night by pain in the head and sacral region, and a bleeding from the ear, lasting three days. This occurred regularly every three weeks. A slight attempt at normal menstruation had showed itself three years previously, after a mustard foot-bath and other remedies. It was accompanied, however, by the bleeding from the ear. The ear shows a large perforation, the breasts are poorly developed, no cervix uteri is discoverable, and considerable leucorrhœa exists.

---

TREATMENT OF CHAPPED HANDS AND FROSTED FEET.—At a meeting of the Philadelphia County Medical Society, held November 21st, 1883, Dr. Carl Seiler called attention to the value of tincture of benzoin in the treatment of chapped hands and frosted feet. He had used it in a number of cases with much success. It is applied by simply painting it on the skin. The stockings may be prevented from sticking to the feet by rubbing some oil over the benzoin.—*Polyclinic.*

---

HOLMES says: "Diagnosis has reached a wonderful degree of accuracy; prognosis has become a terrible kind of second sight which is not always handled carefully enough; treatment gains a little with every decade."—*Ex.*

THE WET PACK IN SCARLET FEVER.—Dr. A. F. Rundell, in *Medical Call*, speaks highly of this remedy. He says he always puts the patient in a wet pack, if called before the rash is thoroughly out, and before desquamation begins to take place, using water at the temperature of 67 to 70 degrees. The patient is left in the pack from one-half to three-quarters of an hour, and is given all the cold water he wants to drink. The pack may be repeated to reduce fever. He finds this treatment to act nicely in allaying fever and bringing out the rash.—*Obstetric Gazette.*

---

A CORRESPONDENT of the *Medical and Surgical Reporter* (March 1st, narrates a case where it seems tolerably certain that scarlet fever was transmitted by means of a letter. At least there is much less room for doubt than in many cases where such a cause is popularly assigned. The outbreak was in a country house half a mile distant from the nearest neighbor, and the family had occupied the house for three years; the children had not been away from the farm for two months, and no one had been in the house who had had the fever or been where it was. In fact, no case of the disease had been known of or heard of by the physician for some months anywhere in the county. It appeared, however, that the mother had received a letter from her brother only a short time before, stating that her brother's family had just lost a child with scarlet fever. This letter contained a photograph. The letter was received only seven days before the first child was taken sick, and the children all handled the letter and picture.—*Boston Medical and Surgical Journal.*

---

LIGATION OF COMMON CAROTID FOR TRAUMATIC ANEURISM.—Dr. J. Thad. Johnson, of Atlanta, reports the case of a man who received a penetrating wound from the blade of an ordinary pocket-knife, in the side of the neck, about an inch below the angle of the jaw. Free hemorrhage occurred, but was soon arrested, and then entirely ceased. On the eighth day a small tumor was discovered, which steadily grew. On the twentieth day hemorrhage again occurred, and was again stopped by pressure. The tumor now grew so rapidly that the common carotid was ligated on the twenty-fourth day. Several hours after the operation the patient was discovered to be hemiplegic. He gradually sank, and died thirty-six hours after the operation.—*Southern Medical Record*, January, 1884.—*Med. News.*

